# Perioperative anaphylaxis attributed to acetaminophen following intravenous acetaminophen administration: a case report

**DOI:** 10.1186/s40981-025-00816-6

**Published:** 2025-09-26

**Authors:** Yasuhiro Amano, Yosuke Taki, Yuko Konishi, Tasuku Fujii, Takahiro Tamura

**Affiliations:** 1 Department of Anesthesiology, Nagoya University Graduate of School of Medicine, 65 Tsurumai-Cho, Showa-Ku, Nagoya, 466-8550 Japan; 2 Department of Ophthalmology, Nagoya University Graduate of School of Medicine, Nagoya, Japan; 3 Endowed Division of Perioperative Management, Nagoya University Graduate School of Medicine, Nagoya, Japan

**Keywords:** Acetaminophen, Perioperative anaphylaxis, Mannitol, Skin tests, Basophil activation test

## Abstract

**Background:**

Anaphylaxis caused by intravenous acetaminophen is extremely rare, but a few case reports have identified mannitol, an excipient, as the causative component. Since mannitol is widely present in medications and foods, distinguishing the true antigen is essential to prevent recurrence.

**Case Presentation:**

A 67-year-old woman developed anaphylaxis with pulseless electrical activity during ophthalmic surgery after intravenous administration of acetaminophen (Acelio®). Allergy testing revealed positive reactions to both Acelio® and acetaminophen in skin tests and the basophil activation test, while reactions with mannitol were negative. Acetaminophen was confirmed as the causative agent. Hence, the patient was instructed to avoid only acetaminophen.

**Conclusions:**

Accurate identification of the causative component in intravenous acetaminophen formulations is critical. Clarifying whether the reaction is due to the active ingredient or an excipient such as mannitol helps prevent unnecessary drug restrictions and expands future treatment options.

## Background

Intravenous acetaminophens (paracetamol) are widely used for perioperative analgesia, and their formulations typically contain excipients. Although anaphylaxis associated with intravenous acetaminophen formulation is rare, a previous case series identified the excipient mannitol as the causative antigen [1]. Since mannitol is commonly present in various medications and foods, determining whether the antigen is the active ingredient or an excipient is essential for ensuring patient safety during future anesthesia and for preventing recurrence of anaphylaxis. Here, we report a case of anaphylaxis that occurred during ophthalmic surgery following intravenous acetaminophen administration. In this case, the causative agent was confirmed to be acetaminophen itself, not mannitol.

### Case presentation

A 67-year-old woman was scheduled to undergo bilateral cataract surgery. Two weeks before the elective surgery, the patient fell and fractured her right clavicle. She received oral acetaminophen for 1 week to relieve the pain from a fracture, and cataract surgery was performed as scheduled. The operation was performed under general anesthesia because the patient had intellectual disabilities. General anesthesia was induced using propofol, rocuronium, fentanyl, and remifentanil. Endotracheal intubation was uneventful, and surgery was initiated after povidone-iodine disinfection of the eyes. The lens capsule was dyed using indocyanine green and prepared with a viscoelastic agent, containing purified sodium hyaluronate and chondroitin sulfate sodium. Hyaluronate was subsequently inserted into the anterior chamber, allowing successful implantation of the intraocular lens without complications. However, 13 min after intravenous acetaminophen (Acelio®, TERUMO, Tokyo, Japan) administration, the patient’s peak airway pressure increased from 15 cmH_2_O to 35 cmH_2_O under volume-controlled ventilation, SpO_2_ dropped to 85%, and systolic blood pressure decreased to 34 mmHg. At this stage, the carotid pulse was palpable. Although the anesthesiologist repeatedly administered phenylephrine and 50 μg of norepinephrine, the carotid pulse was faintly palpable 10 min later, and the event was considered as pulseless electrical activity. Chest compressions were immediately initiated, and additional personnel were called to assist; preparations for adrenaline administration were made. A pulse check performed 1 min later revealed return of spontaneous circulation. A systemic rash was observed, and anaphylaxis was suspected, leading to the initiation of continuous adrenaline infusion in addition to the ongoing norepinephrine infusion, which resulted in stabilization of the blood pressure. Cataract surgery on the other eye was aborted, and the patient was transferred to the surgical intensive care unit under intubation with sedation.

**Fig. 1:**
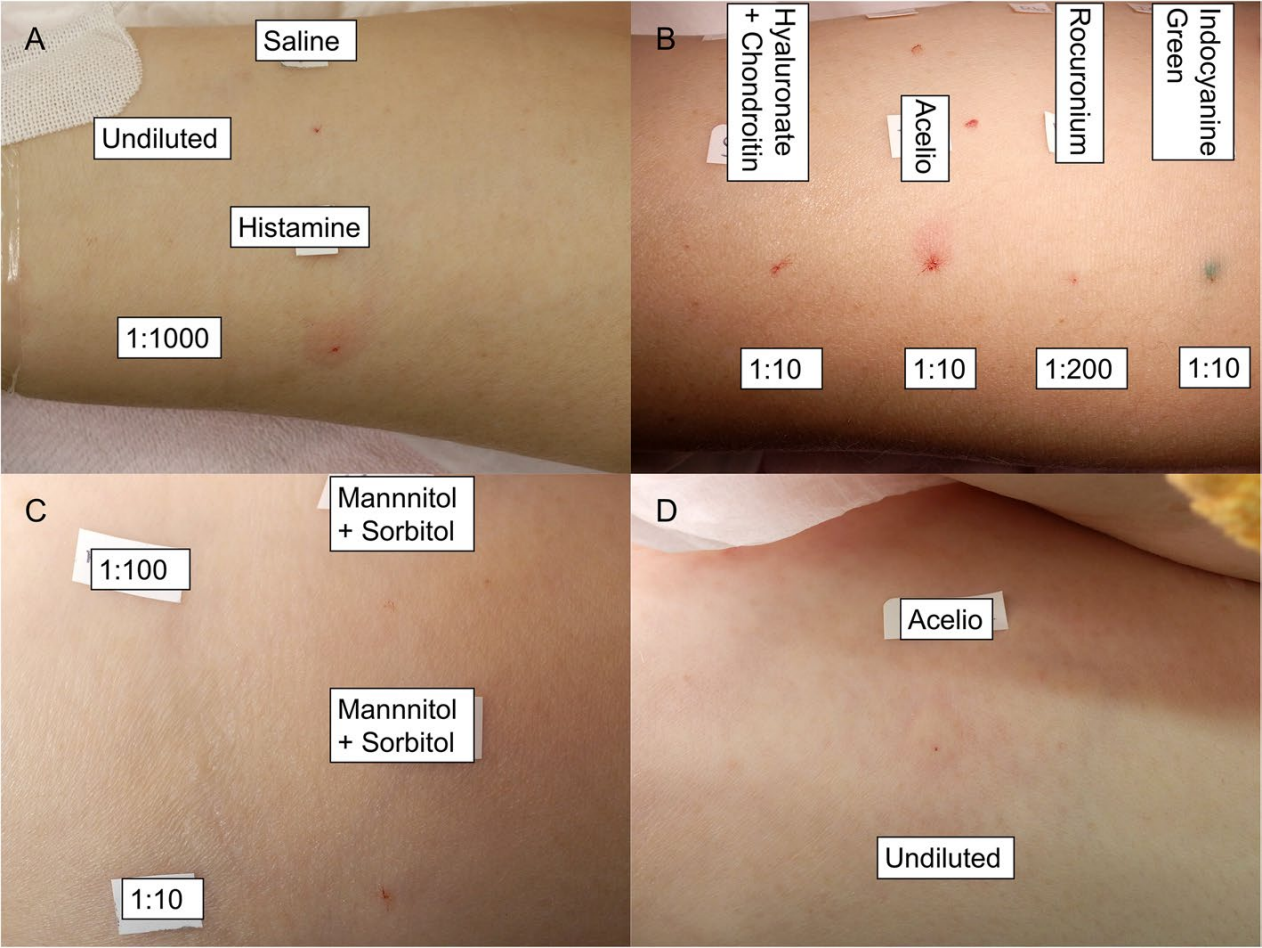
Intradermal tests results. The names and concentration for each drug used in the intradermal tests are shown. **A**: Intradermal tests with positive and negative control at the left forearm. **B**: Intradermal tests with the four suspected causative drugs at suggested intradermal tests concentration at left forearm. **C**: Intradermal tests with Mannitol + Sorbitol at left upper arm. **D**: Intradermal tests with undiluted Acelio at left upper arm

**Fig. 2:**
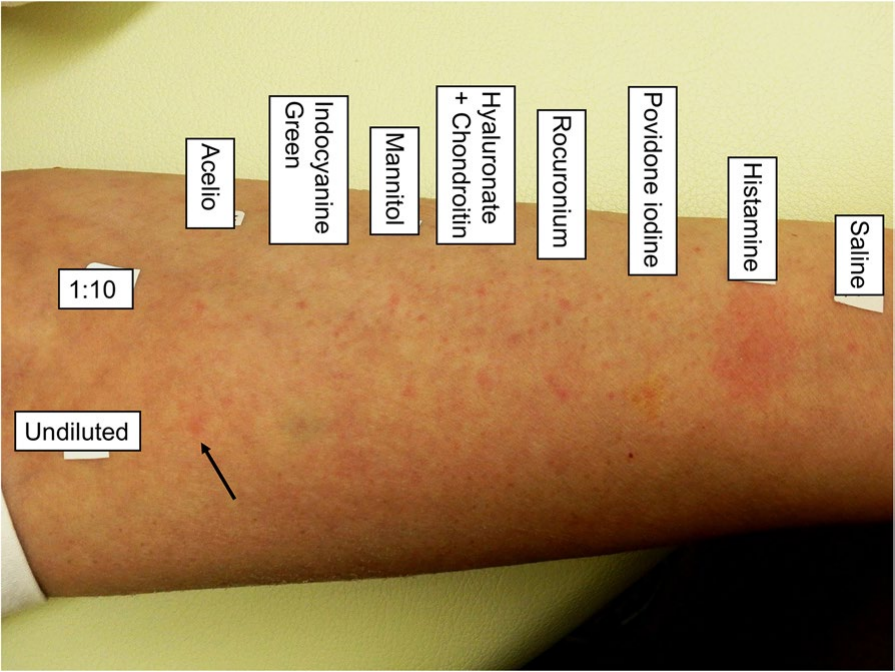
Skin prick tests results. The black arrow indicates the wheal formed after the prick test with undiluted Acelio

**Fig. 3:**
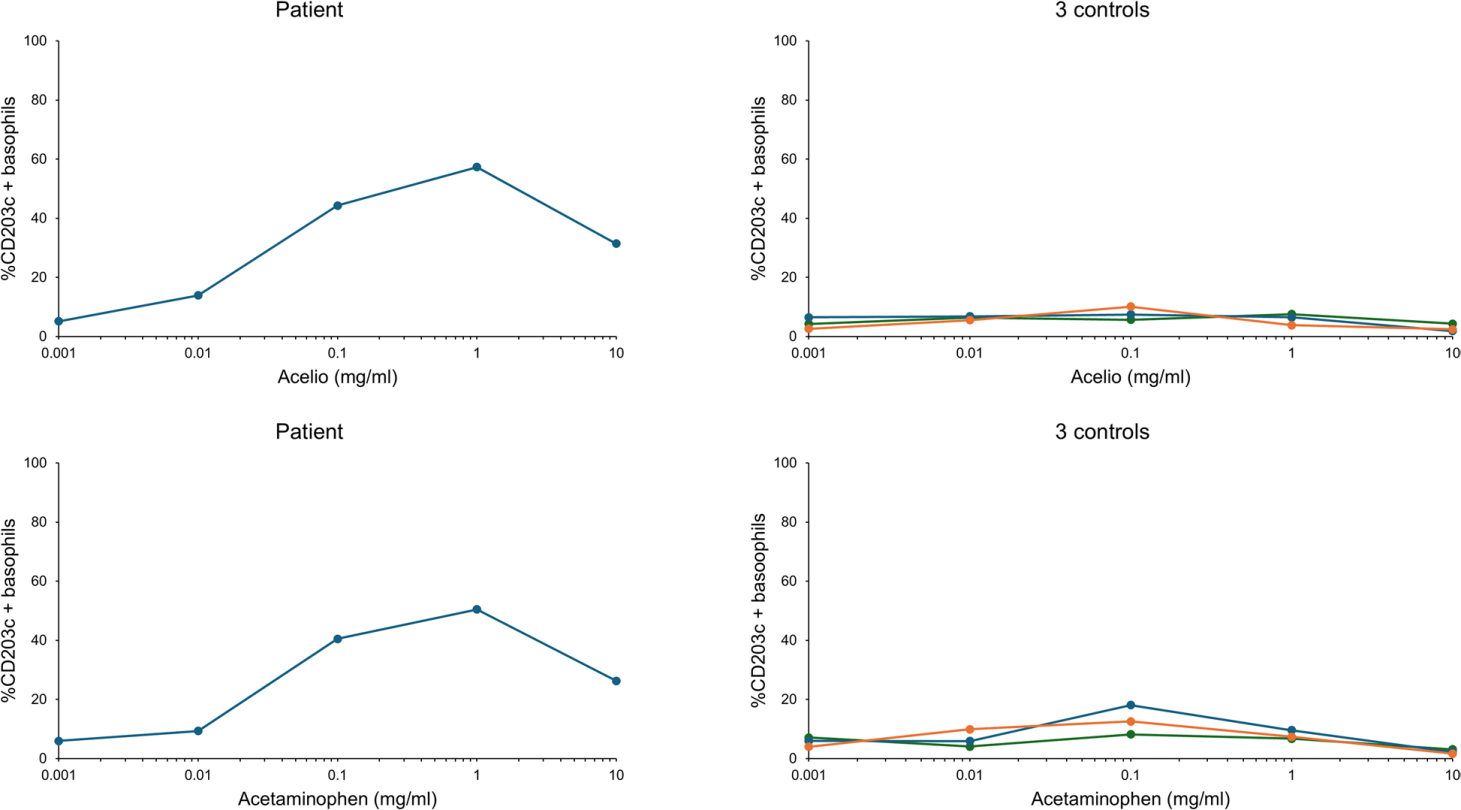
Results of basophil activation tests with CD203 of the patient and 3 controls with five serial dilutions of Acelio and acetaminophen

An intradermal test (IDT) was performed under sedation in the surgical intensive care unit on the day of the reaction. As mannitol was unavailable at our hospital that day, a mannitol/sorbitol formulation was used instead. All drugs yielded negative results in two dilutions, and only the undiluted Acelio yielded positive results (Table 1, Fig. 1) [2]. The patient was extubated the day after onset and discharged with no sequelae the following day. We also performed a skin prick test (SPT) 44 days after the reaction. Undiluted Acelio yielded positive results, whereas the other drugs yielded negative results (Table 2, Fig. 2) [3]. We performed a basophil activation test (BAT) to identify the causative components. An Allergenicity kit (Beckman Coulter, Brea, CA, USA) was used for the BAT. Acelio, acetaminophen (p-acetamidophenol; NACALAI TESQUE, Kyoto, Japan), and mannitol were diluted with normal saline to obtain five serial dilutions (1, 1:10, 1:100, 1:1000, and 1:10,000). BAT methods are detailed elsewhere [4]. The patient’s CD203c basophils demonstrated a concentration response curve with increasing Acelio and acetaminophen concentrations (Fig. 3). The patient yielded negative BAT results for mannitol. We also performed BAT for Acelio and acetaminophen in three healthy controls to ensure specificity. All the three controls yielded negative results (Fig. 3). Plasma histamine and tryptase were significantly elevated to 7.69 μg/l and 97.6 μg/l, respectively, 30 min after symptom onset compared to the levels measured 24 h after the onset (0.47 μg/l and 9.5 μg/l) [5, 6]. Hence, acetaminophen-induced anaphylaxis was confirmed based on positive diagnostic tests, and the patient was advised to avoid only acetaminophen-containing drugs. Surgery of the other eye was not performed owing to the withdrawal of consent by the patient’s family.

**Table 1:** Intradermal test results

Drug	Dilution	Wi (mm)	W20 (mm)	Flare (mm)	Judgement
Saline 9 mg/ml	Undiluted	3.0	0	0	-
Histamine 10 mg/ml	1:1000	3.0	9.4	15.8	+
Hyaluronate 30 mg/ml + Chondroitin 40 mg/ml	1:10	4.0	0	0	-
	1:100	3.6	0	0	-
Acelio (Acetaminophen 10 mg/ml + Mannitol 38.5 mg/ml)	Undiluted	4.1	8.6	21.6	+
	1:10	3.7	7.2	0	-
	1:100	4.4	3.9	0	-
Rocuronium 10 mg/ml	1:200	4.0	0	0	-
	1:2000	3.3	0	0	-
Indocyanine Green 5 mg/ml	1:10	3.8	4.0	0	-
	1:100	3.7	0	0	-
Mannitol 150 mg/ml + Sorbitol 50 mg/ml	1:10	3.7	0	0	-
	1:100	4.5	0	0	-

*Wi* diameter of the initial wheal just after injection, *W20* diameter of the wheal 20-min post injection

Saline and histamine were used as the negative and positive controls, respectively. Saline was used for drug dilution. The injection volume for the intradermal test was 0.02 ml for all drugs and a 3–5 mm wheal was made at the patient’s forearm. The diameters of the wheals and flares were measured using digital calipers. A positive reaction was made based on the following criteria [2]: W20 ≥ Wi + 3 mm with surrounding flare. After confirming the positive result in the control, two procedures of intradermal tests with two dilutions of four suspected drugs were performed. A 10-fold dilution of Acelio resulted with a wheal enlargement of more than 3 mm; however, there was no surrounding flare, making the result uncertain. Therefore, an additional intradermal test was performed using mannitol 150 mg/ml + sorbitol 50 mg/ml, and undiluted Acelio. Undiluted Acelio met the criteria for a positive reaction

**Table 2:** Skin prick test results

Drug	Dilution	Wheal (mm)	Judgement
Saline 9 mg/ml	Undiluted	0	-
Histamine 10 mg/ml	Undiluted	6.6	+
Acelio (Acetaminophen 10 mg/ml + Mannitol 38.5 mg/ml)	Undiluted	3.5	+
	1:10	0	-
Rocuronium 10 mg/ml	Undiluted	0	-
	1:10	0	-
Indocyanine Green 5 mg/ml	Undiluted	0	-
	1:10	0	-
Povidone iodine 100 mg/ml	Undiluted	0	-
	1:10	0	-
Mannitol 200 mg/ml	Undiluted	0	-
	1:10	0	-

Saline and histamine were used as the negative and positive controls, respectively. Saline was used for drug dilution. The diameters of the wheals were measured using digital calipers. A positive reaction was made based on the following criteria: wheal ≥ 3 mm [3]. Only undiluted Acelio showed positive results

## Discussion

Patients with suspected perioperative anaphylaxis should be investigated to identify the culprit drug and safe alternatives, after excluding the differential diagnoses [2, 7]. Although vasovagal reflex can occur during ophthalmic surgery, there was no evidence of eye compression. The patient developed multiple-organ symptoms and elevated histamine and tryptase levels. Recently, the hypersensitivity clinical scoring scheme was developed as an objective tool for assessing the likelihood of anaphylaxis [8]. The patient’s score was 39, indicating a high probability of anaphylaxis. To identify the causative drug of perioperative anaphylaxis, SPT should be performed first, followed by IDT [2]. However, owing to subcutaneous bruising and swelling throughout the right arm, caused by the right clavicle fracture, only the patient’s left arm could be used for the skin tests. Although performing skin testing on the back under sedation is technically possible, the Glasgow Coma Scale score was E1VTM4; thus, we considered it invasive and uncomfortable for the patient to be placed in the prone position with the right upper arm immobilized by a band and intubated during the procedure. IDT has a higher sensitivity than SPT for identifying the causative agent of perioperative anaphylaxis; however, IDT is more painful [9]. Because the patient became highly agitated during preoperative blood sampling, we considered IDT in the outpatient setting after a 4–6-week interval was challenging [10]. Therefore, we performed the IDT under sedation on the day of the reaction, fully aware of the risk of false negatives. Although a higher concentration of drugs can cause irritation and false-positive results in IDT, a previous study reported that IDTs performed on healthy individuals using 100 mg/ml acetaminophen yielded negative results [11]. Since the Acelio concentration used in IDT in our patient was ≤ 10 mg/ml, our result was unlikely to be false positive. The positive SPT results also suggested that Acelio was the culprit drug.

Identifying the causative component is advantageous for both patients and healthcare providers. Mannitol, which is often added as an excipient to intravenous acetaminophen products, is widely used in pharmaceutical and food products. Labeling both acetaminophen and mannitol as antigens reduces the number of available medications for healthcare providers and places a significant burden on patients by increasing the number of drugs and food products to be avoided in daily life. Although mannitol yielded negative results in the IDT and SPT, this does not necessarily rule out mannitol as the antigen. Mannitol has been reported to occur naturally in pomegranate and mushrooms [12]; however, inquiries with the patient's family did not clarify whether the patient had any known hypersensitivity to these substances. Since pure acetaminophen was only available for research purposes, we performed BAT for Acelio, acetaminophen, and mannitol. One of the advantage of the BAT is its ability to utilize drugs that are otherwise restricted for research use only. Contrary to previous reports [1], Acelio and acetaminophen yielded positive results in the BAT. Drug provocation tests with Acelio and mannitol could enable definitive identification of the antigen. However, considering the patient’s life-threating anaphylaxis, the risk outweighs the benefits, and we considered drug provocation tests as unnecessary [13]. We could not determine the immunological pathway since no reagent assessing acetaminophen-specific IgE is commercially available in Japan. BAT using wortmannin, a specific inhibitor of phosphoinositide 3-kinase, may help determine whether acetaminophen-induced anaphylaxis is mediated by an IgE-dependent immune response [14, 15]. Finally, mannitol allergy was delabeled, and the patient was instructed to avoid acetaminophen only. We assumed that the patient had taken oral acetaminophen for 1 week following a clavicle fracture, became sensitized approximately 2 weeks after the initial dose, and subsequently developed anaphylaxis upon intravenous administration of acetaminophen during cataract surgery [16].

Intravenous acetaminophen administration can cause life-threatening anaphylaxis. Identifying the causative components of intravenous acetaminophen benefits both healthcare providers and patients by increasing the choice of medications available for use.

## Data Availability

Data relevant to this case report are not publicly available because of concerns regarding patient privacy but are available from the corresponding author upon reasonable request.

## Abbreviations

IDT: Intradermal test
SPT: Skin prick test
BAT: Basophil activation test
NSAIDs: Nonsteroidal anti-inflammatory drugs

## References

[1] Jain SS, Green S, Rose M. Anaphylaxis following intravenous paracetamol: the problem is the solution. Anaesth Intensive Care. 2015;43:779–81. 26603804 10.1177/0310057X1504300617

[2] Garvey LH, Ebo DG, Mertes PM, Dewachter P, Garcez T, Kopac P, et al. An EAACI position paper on the investigation of perioperative immediate hypersensitivity reactions. Allergy. 2019;74:1872–84. 30964555 10.1111/all.13820

[3] Mertes PM, Malinovsky JM, Jouffroy L, Aberer W, Terreehorst I, Brockow K, et al. Reducing the risk of anaphylaxis during anesthesia: 2011 updated guidelines for clinical practice. J Investig Allergol Clin Immunol. 2011;21:442–53. 21995177

[4] Horiuchi T, Yokohama A, Orihara M, Tomita Y, Tomioka A, Yoshida N, et al. Usefulness of basophil activation tests for diagnosis of Sugammadex-induced anaphylaxis. Anesth Analg. 2018;126:1509–16. 29517573 10.1213/ANE.0000000000002879

[5] Valent P, Akin C, Arock M, Brockow K, Butterfield JH, Carter MC, et al. Definitions, criteria and global classification of mast cell disorders with special reference to mast cell activation syndromes: a consensus proposal. Int Arch Allergy Immunol. 2012;157:215–25. 22041891 10.1159/000328760 PMC3224511

[6] Horiuchi T, Takazawa T, Haraguchi T, Orihara M, Nagumo K, Saito S. Investigating the optimal diagnostic value of histamine for diagnosing perioperative hypersensitivity: a prospective, observational study. J Anesth. 2023;37:645–9. 37156974 10.1007/s00540-023-03199-z

[7] Takazawa T, Yamaura K, Hara T, Yorozu T, Mitsuhata H, Morimatsu H; et al. Practical guidelines for the response to perioperative anaphylaxis. J Anesth 2021;35:778–93. 10.1007/s00540-021-03005–8. 34651257

[8] Hopkins PM, Cooke PJ, Clarke RC, Guttormsen AB, Platt PR, Dewachter P, et al. Consensus clinical scoring for suspected perioperative immediate hypersensitivity reactions. Br J Anaesth. 2019;123:e29-37. 31029409 10.1016/j.bja.2019.02.029

[9] Gomes ER, Brockow K, Kuyucu S, Saretta F, Mori F, Blanca-Lopez N, et al. Drug hypersensitivity in children: report from the pediatric task force of the EAACI Drug Allergy Interest Group. Allergy. 2016;71:149–61. 26416157 10.1111/all.12774

[10] Soetens F, Rose M, Fisher M. Timing of skin testing after a suspected anaphylactic reaction during anaesthesia. Acta Anaesthesiol Scand. 2012;56:1042–6. 22313451 10.1111/j.1399-6576.2011.02643.x

[11] Galindo PA, Borja J, Mur P, Feo F, Gómez E, García R. Anaphylaxis to paracetamol. Allergol Immunopathol (Madr). 1998;26:199–200. 9816409

[12] Hegde VL, Venkatesh YP. Anaphylaxis to excipient mannitol: evidence for an immunoglobulin E-mediated mechanism. Clin Exp Allergy. 2004;34:1602–9. 10.1111/j.1365-2222.2004.02079.x. 15479277 10.1111/j.1365-2222.2004.02079.x

[13] Garvey LH, Ebo DG, Krøigaard M, Savic S, Clarke R, Cooke P, et al. The use of drug provocation testing in the investigation of suspected immediate perioperative allergic reactions: current status. Br J Anaesth. 2019;123:e126–34. 10.1016/j.bja.2019.03.018. 31027914 10.1016/j.bja.2019.03.018

[14] Horiuchi T, Takazawa T, Sakamoto S, Orihara M, Yokohama A, Uchiyama M, et al. Possible immunoglobulin-E-dependent sugammadex-induced anaphylaxis caused by an epitope other than γ-cyclodextrin: a case report. J Med Case Reports. 2021;15:1–4. 10.1186/s13256-021-02894-3. 10.1186/s13256-021-02894-3 PMC8178030 34088358

[15] Orihara M, Takazawa T, Horiuchi T, Nagumo K, Maruyama N, Tomioka A, et al. Intraoperative chlorhexidine-induced anaphylaxis suggesting an immunoglobulin-E-dependent mechanism indicated by basophil activation tests: two case reports. JA Clin Rep. 2022;8:91. 10.1186/s40981-022-00581-w. 36417006 10.1186/s40981-022-00581-w PMC9684359

[16] Chen Q, Xie M, Liu H, Dent AL. Development of allergen-specific IgE in a food-allergy model requires precisely timed B cell stimulation and is inhibited by Fgl2. Cell Rep. 2022;39:110990. 10.1016/j.celrep.2022.110990. 35767958 10.1016/j.celrep.2022.110990 PMC9271337

